# Long-Term Efficacy and Complications of Radiofrequency Thermocoagulation at Different Temperatures for the Treatment of Trigeminal Neuralgia

**DOI:** 10.1155/2020/3854284

**Published:** 2020-03-04

**Authors:** Tao Hong, Yuanyuan Ding, Peng Yao

**Affiliations:** Department of Pain Management, Shengjing Hospital of China Medical School, No. 36, Sanhao Street, Heping District, Shenyang 110004, China

## Abstract

Trigeminal neuralgia (TN) is a common neuropathic pain that seriously affects the daily life of patients. Many invasive treatments are currently available for patients who respond poorly to oral carbamazepine or oxcarbazepine. Among them, radiofrequency (RF) treatment is a viable option with reliable initial and long-term clinical efficacy. The long-term analgesic effects of radiofrequency thermocoagulation (RFT) at high temperatures (≥80°C) are not superior to those at relatively low temperatures (60–75°C). In contrast, the higher the temperature, the greater the risk of complications, especially facial numbness, masticatory muscles weakness, and corneal hypoesthesia. Some patients even experience irreversible lethal complications. Therefore, we recommend low-temperature RFT (60–75°C) for treatment of TN. The therapeutic effects of pulsed radiofrequency (PRF) are controversial, whereas PRF (≤75°C) combined with RFT can improve long-term effects and decrease the incidence of complications. However, large-scale clinical trials are needed to verify the efficacy of the combination of PRF and RFT.

## 1. Introduction

Trigeminal neuralgia (TN) is a common neuropathic pain disorder with symptoms of transient, electric-shock-like pain affecting one or more branches of the trigeminal nerve. Talking, eating, brushing teeth, and slight touching of trigger point located in the oral or perioral region can induce severe and brief pain. Severe pain can affect the daily activities of patients. Those who experienced long-term pain often experience emotional changes, such as anxiety and depression. Epidemiological studies have shown that elderly women have a higher incidence than men (1.5–2.1 times more than men), and the annual prevalence of the disease is 12.6–28.9 cases per 10 million people [1–4]. Patients with severe TN who cannot be completely controlled by oral carbamazepine or oxcarbazepine or who are intolerant to the adverse effect of drugs can benefit from invasive surgery. Common surgical interventions include percutaneous balloon compression [5], microvascular decompression (MVD) [6], gamma knife radiosurgery [7], RFT [8, 9], PRF [10, 11], and intradermal and/or subcutaneous injections of Botox [12], intragasserian phenol glycerite [13], and peripheral alcohol [14].

Although there are some serious complications reported in prior studies [9, 15–20], RFT is still an effective treatment for TN that can instantly relieve pain in 90%–100% of cases [15, 17–19, 21–24]. Kanpolat et al. have observed 1561 patients who underwent single-procedure RFT [21]. The proportion of patients who were painless for 5 years and did not require oral medication was as high as 57.7%. By 15 years, 42.2% of patients still had no recurrence of pain. Fouad observed 312 patients with TN [25]. The rate of pain relief after 2 years of RFT was as high as 97.2%, and the rate of pain recurrence tracking to 8.8 years was only 7.6%. Tang et al. have observed 1161 patients who underwent RFT via the Hartel anterior approach, and the pain relief rate was as high as 52% after 11 years [9]. The purpose of this review is to summarize the different temperatures utilizing RFT treatment for TN. Thus, we hope to reveal the optimal temperature suitable in RFT operation with best outcome.

## 2. Principles of RF

The main principle of RF treatment of TN is blockage pain signal conduction by high temperature up to 90°C to destroy the nerve (RFT) or modulation of the nociceptive nerve function of trigeminal nerve with temperature not exceeding 42°C (PRF). Two sets of technology can reduce the transmission of peripheral stimuli to the central nervous system, thereby preventing the disastrous pain. Although the pathogenesis of idiopathic trigeminal neuralgia is not completely clear, most scholars tend to believe that TN was associated with arterial or venous vascular compression, strategically located at the root entry zone. They recommended that MVD was the gold standard treatment for refractory TN and did not consider RF treatment to be involved in this pathogen [26, 27]. Prior clinical reports indicated that MVD can achieve similar or better results than RFT of the gasserian ganglion or trigeminal rootlets [19, 28, 29]. However, RFT is an alternative treatment for patients with recurrent TN after MVD, and it achieved good pain relief condition [23]. Therefore, by interfering with the function of the trigeminal nerve or destroying the integrity of its anatomical structure, therapeutic effects can be achieved.

## 3. The Pain Relief Rate of RFT at Different Temperatures for TN Treatment

There is no specific standard for temperature selection in RFT. A review of the published English literature indicated that the temperature for RFT varied widely among studies (60°C to 95°C). The theoretical basis for choosing high-temperature RFT is the hypothesis that the recurrence rate of TN is correlated with the degree of postoperative sensory deficit [9, 22, 30]. In support of this possibility, Taha et al. [22] have reported that the recurrence rate within 4 years was 100% among patients with mild postoperative facial numbness, whereas the rate was only 10% over a period of 10 years among patients with dense hypalgesia. Notably, those with analgesia had the lowest recurrence rate within 15 years. In addition, previous animal experiments have reported that temperatures below 80°C can selectively damage the Aб and C unmyelinated nerve fibers without damaging the A*α* and A*β* nerve fibers, thereby blocking pain transmission without affecting facial tactile sensation [31].

High-temperature RFT (≥75°C) often leads to serious complications, such as severe facial numbness (84.7–100%) [9, 25], ptosis (0.7%) [9], keratitis (1.94–4.9%) [16, 25], corneal ulcers (0.6–2.6%) [9, 21], diplopia (0.66%) [9], abducens nerve damage (0.66–0.8%) [15, 21], transient vision loss and blindness (0.86%) [15], mandibular deviation (4%) [15], masticatory muscle weakness (8–15.8%) [9, 16], hearing loss (0.4%) [9], cerebrospinal fluid leakage (0.17%) [9], and even death (1.8–4%) [9, 32]. Those complications would cause pain to patients and lead to disputes between doctors and patients. The pain relief rate of RFT at ≥80°C had no significant advantage over RFT at 60–75°C. Kosugi et al. have reported the use of RFT at 90°C for patients with pain at the V2 and/or V3 branch, which achieved 1- and 2-year painlessness rates of 40.5%–80.2% and 17.1%–54.9%, respectively [30]. Son et al. have reported that after using 80°C RFT to treat TN, the proportions of patients achieving BNI I and II within 38 months were 71% and 15.8%, respectively [33].

Some studies use the recurrence rate as an indicator to assess the efficiency of high-temperature RFT for TN. The percentage of patients with recurrent pain ranged from 7.8% to 42.7% with tracking from 11.6 to 15 years [15, 21]. However, there were some limitations to the studies. The studies did not provide a detailed definition of “pain relief” or specify whether the patients used medication to control pain after the procedure. In addition, they included patients with secondary trigeminal neuralgia (STN) who had received other invasive interventions without success. Nonetheless, high-temperature RFT was not superior to low-temperature RFT in terms of the long-term pain relief rate. Tang et al. have analyzed a total of 1161 patients retrospectively and divided them into 65–70°C, 75°C, and 80–85°C groups according to the temperature used in the procedure [9]. Notably, there was no significant difference in the long-term pain relief rate among the three groups, thus suggesting that a higher temperature for RFT would not further increase long-term pain relief, and a temperature >65°C can generate satisfactory analgesic effects.

To explore the optimal temperature for achieving efficacious treatment of RFT, we conducted a multicenter clinical observational study to evaluate the long-term efficacy of low-temperature RFT, including temperatures of 62°C, 65°C, 68°C, 70°C, and 75°C for RFT [16, 34]. Patients with STN or V1 division of TN were excluded, as well as those who did not respond to other invasive procedures. The probability of experiencing no pain and requiring no drugs (BNI I) was 94.2%, 98.3%, 98.8%, 98.4%, and 98.9% at discharge; 83.8%, 90.1%, 91.4%, 94.3%, and 94.4% at 1 year after the procedure; and 66.7%, 80.5%, 88.2%, 84.3%, and 87.9% at 3 years after the procedure. These data suggest that the pain relief rates at discharge were similar at temperatures from 62°C to 75°C. With a prolonged follow-up time, the pain relief rate in the 62°C and 65°C groups decreased more significantly than that in the 68–75°C groups. However, up to 5 years after procedure, 59% and 64.3% of patients were pain-free in the 62°C and 65°C groups, confirming that 62–65°C in RFT is an efficacious temperature. Yang et al. have achieved a satisfactory pain reduction rate with RFT at 60°C; the proportion of patients whose pain reduction was >90% during 3-month–3.6-year follow-up period was as high as 80%, thus further supporting the efficacy and feasibility of low-temperature RFT [35]. In long-term follow-up, patients in the 75°C group appeared to have higher pain-free rates than those in the 68°C and 70°C groups, but this difference was not statistically significant. Zhao et al. have also compared the effectiveness of RFT at 70°C and 75°C and have found no significant differences in the rates of excellent relief in the Kaplan–Meier actuarial curve [32].

In summary, no significant differences were observed in the rates of pain relief after RFT at ≥80°C and ≤75°C. The probability of excellent pain relief at temperatures of 60–65°C was inferior to that at 68–75°C, whereas the analgesic effects were similar at 70°C and 75°C in some studies. Hence, we recommend RFT at 68–75°C.

## 4. Occurrence of Adverse Effects at Different Temperatures for RFT

The most common adverse effects after RFT treatment include facial numbness, masseter muscle weakness, and decreased or absent corneal reflexes (see Table 1).

The short-term incidence of mild facial numbness after RFT treatment is reportedly 85%–100% [15, 20, 25, 32, 34, 36, 41, 42], and facial numbness gradually decreases or disappears completely within 1 month. Long-term moderate facial numbness (BNI III) mainly occurs at temperatures ≥65°C. More specifically, the incidence was 0.23%–3.19% in the 65–70°C group [36], 24.2% in the 75°C group [34], and 39.8%–97.4% in the ≥85°C group [9, 15]. Severe facial numbness (BNI IV) mainly occurs at temperatures ≥75°C, with an incidence of 3.2% at 75°C [34] and 5.3%–14.5% at ≥80°C [9, 17].

Masticatory muscle weakness is usually associated with damage to the V3 branch (motor nerve), which is most likely to occur at temperatures ≥65°C. The incidence rate is 0.22%–4.8% at ≤68°C [34, 36], with a recovery time of approximately 6 months, 7.1%–12% at 70°C [37, 42], and 25.81%–44.12% at 75°C [34], with a recovery time of more than 1 year. Case reports have described permanent masseter muscle weakness or dysfunction [25].

Anatomically, the V1 division of the TN is adjacent to the oculomotor nerve, trochlear nerve, and abducens nerve. When higher temperatures are used in RFT, the coagulum size due to the radiofrequency probe increases in both length and diameter [43], thus potentially damaging the nerves near TN and causing severe complications, such as ptosis, limited eye movement, diplopia, and corneal ulceration. Therefore, the temperatures associated with V1 division should be selected with caution. Several studies have observed the specific temperature delivered to the V1 division. The incidence of decreased corneal reflex has been found to be 3.57%–17.5% at temperatures of 62–68°C [34, 38]. However, there are no reports of the disappearance of corneal reflexes. When the V1 division combined with V2 or/and V3, a higher stepped temperature from 70°C to 85°C was delivered to the V2 or/and V3. Decreased corneal reflexes were observed in a greater proportion of individuals, at 15.69%–26.47% [20, 34, 38]. Moreover, ptosis, limited eye movement, diplopia, and corneal ulceration have been reported in the literature with temperatures of 70–90°C [9, 21, 23, 44].

In summary, the incidence of adverse effects gradually increases with increasing RFT temperature. As the observation time increases, the degree of these adverse effects gradually lessens, but the recovery times vary with the temperature used for RFT. The incidence and severity of complications are lower at temperatures <70°C, and the recovery time is within 6 months. Severe facial numbness, permanent masticatory atonia, and corneal hypoesthesia are associated with using temperatures at ≥70°C, and some patients had not recovered by the end of the 5-year follow-up. Notably, irreversible and severe complications have low occurrence rates at temperatures ≤65°C for RFT. The probability of complications at 75°C is three- to eightfold greater than that at 70°C. These data suggest that inappropriately increasing the temperature for RFT can damage not only the Aб and C unmyelinated nerve fibers but also the A*α* and A*β* fibers in a nonselective manner, thereby inducing severe complications [31], such as abducens nerve injury, diplopia, and vision loss at 95°C [15], as well as diplopia, hearing loss, and ptosis at 85°C [9].

## 5. The Pain Relief Rate of RFT Combined with PRF for the Temperature of TN

The magnetic field generated by PRF plays a therapeutic role by modulating the release of immune inflammatory mediators or inhibiting C-fiber activation and synaptic transmission [45]. PRF is effective for the treatment of neuropathic pain. Therefore, some researchers believe that PRF can produce good analgesic effects [20, 32, 36, 39, 40, 46–49]. However, the effectiveness of PRF for the treatment of TN remains controversial. The painlessness rate of the treatment is 0%–85.7% for 6 months and 0%–78.6% for 2 years. Luo et al. have explored the causes of the poor effects of PRF and have found that a higher output voltage and electrical field intensity result in better outcomes [50]. Chua et al. have suggested that shortening the pulsed width to 10 ms, enhancing the frequency to 4 Hz, and prolonging the treatment duration to 6 min can improve the long-term analgesic effect, at least to some extent [51].

RFT combined with PRF is a relatively new method for the treatment of TN, especially for the V1 branch. This procedure (RFT at 62–75°C combined with PRF at 42°C) can increase long-term efficacy (85%–92% effective rate for 1 year and 70%–92% for 2 years), while simultaneously minimizing the incidence of adverse effects [32, 36, 39], although there are some opposing opinions [20]. Nonetheless, large-scale clinical trials are needed to evaluate the effectiveness of RFT combined with PRF for the treatment of TN.

## 6. Improving the Effectiveness and Feasibility of RFT

Increasing the temperature is not the only effective way to improve the efficacy of RFT treatment. Regulating the distance between the tip of the needle and the target nerves is crucial. The target location should be in the junction between the third division and the trigeminal ganglion, which can effectively improve the postoperative effects [15]. In addition, three-dimensional computed tomography (CT) or neuronavigator-guided control can be used to improve the accuracy of the puncture by monitoring the direction and angle of the needle in real time [52, 53]. This technology simplifies the puncture process and avoids severe intraoperative or postoperative side effects, such as damage to the optic nerve, auditory nerve, internal carotid artery, and cavernous sinus. It is especially important to select the parameters of RFT. For example, at <0.1 V, an abnormal sensation or muscle contraction is induced in the areas controlled by the target nerves, suggesting that the needle tip is located in the nerve sheath. When RFT is performed, A*α* and A*β* fibers are destroyed nonselectively, rather than just the Aб and C unmyelinated nerve fibers. Therefore, we recommended refraining from using high temperatures for RFT. If the above signs appear at 0.1–0.3 V, the needle tip should be directed to the nerve root, and the temperature should be controlled to ≤70°C. At >0.3 V, we suggest that the needle tip be at a certain distance from the nerve and be located near the ganglion. Consequently, only the small nerves (i.e., the Aб and C unmyelinated nerve fibers) will be heated, and the location of the needle tip should be adjusted to enhance efficiency [9, 37].

## 7. Conclusion

In summary, RF treatment is a safe, effective, and minimally invasive procedure of TN. An optimal temperature should be selected according to the branches of the trigeminal nerves involved in the RFT procedure. If V1 is involved, in order to decrease the risk of corneal reflex weakness or disappearing, a temperature of ≤65°C is recommended. If V2 and/or V3 are involved, 65–70°C temperatures are more suitable to effectively control pain and decrease the probability of adverse reactions. Too high temperature (≥80°C) should be avoided because it could seriously damage the tissue and is associated with irreversible complications. In order to improve the accuracy of the puncture and decrease the complications of injuring crucial blood vessels and nerves caused by repeated punctures, it is better to use three-dimensional CT or neuronavigator as guidance. Although the value of PRF for the treatment of TN remains controversial, RFT at ≤75°C combined with PRF is a feasible option, and further clinical trials are required to evaluate efficacy.

## Figures and Tables

**Table 1:** Basic information, intervention procedure parameters, outcomes, and the rate of adverse effects of included observational studies.

First author (year)	Country	Sample	TN type	M/F	Target nerve stimulation	Temperature	Clinical setting	Follow-up time	Outcome	The rate of complications
Tang [9] (2016)	China	1161	ITN	462/675	2 Hz, ≥ 2 V, 50 Hz, ≤ 0.5 V	65–85°C	Retrospective cohort study	46 ± 31 months	The optimal RFT temperature to maximize pain relief and minimize facial numbness or dysesthesia may be 75°C	Masseter muscle weakness (8%), corneitis (2.6%), diplopia (1%); low pressure headache (0.2%), ptosis (0.7%); difficulty with mouth opening (0.4%); hearing loss (0.4%)

Kanpolat [21] (2001)	Turkey	1600	ITN	766/834	50 Hz, 0.2 to 1 V	55–70°C	A single-arm retrospective analysis	68.1 ± 66.4 months	RFT has a high long-term success rate	Corneal reflex diminish (5.7%), masseter weakness and paralysis (4.1%), dysesthesia (1%), anesthesia dolorosa (0.8%), keratitis (0.6%), permanent cranial nerve VI palsy (0.12%), cerebrospinal fluid leakage (0.12%), carotid-cavernous fistula (0.06%), aseptic meningitis (0.06%)

Taha [22] (2016)	US	154	ITN	54/100	NA	NA	A single-arm retrospective study	15 years	RFT has a high long-term success rate. Patients with mild hypalgesia had the highest pain recurrence rate	Analgesia (46%), dense hypalgesia (42%), mild hypalgesia (12%), decreased corneal reflex (13.63%), absent corneal reflex (5.19%), keratitis (1.94%), developed masseter weakness (14.28%)

Fraioli [15] (2009)	Italy	158	ITN + STN	NA	NA	90 to 95°C, 10 min	A single-arm retrospective study	Median period of 11.6 years	RFT is immediately effective, low rate of recurrence procedure	Unwanted 1st and 2nd division hypoanesthesia (1.27%), paresthesias requiring transient medical treatment (3.80%), masseter dysfunction (3.80%), transient 6th nerve palsy (0.63%)

Fouad [25] (2011)	Egypt	312	ITN + STN	124/188	0.1–0.3 V at 50–75 Hz, for 3 ms	60°C, 120 s, for ITN	Retrospective analysis	7 years	The outcome depends on the type of TN with best results with classical idiopathic type. Also better results occurred with isolated V3 branch	Minor dysesthesia (28%), major dysesthesia (8%), temporary corneal anesthesia (0.96%), permanent trigeminal motor dysfunction (0.16%), meningitis (0.32%)

Yao [16] (2016)	China	62	ITN	27/35	50 Hz, 1 ms, 0.1–0.2	68°C, 180 s; 75°C, 180 s	Prospective cohort study	5 years	The rate of pain relief after treatment at 75°C was slightly higher than at 68°C. The incidence and severity of complications were greater at 75°C, and therefore the patient satisfaction at the higher temperature was lower	Facial numbness at 68 and 75°C accounted for 12.9%, 75.8%; corneal hypoesthesia accounted for 1.6%, 19.35%; masticatory atonia accounted for 4.8%, 25.8%

Zhao [32] (2015)	China	80	ITN	34/46	NA	70°C, 120 s; 70°C, 120 s; + 42°C, 240 s; 75°C, 120 s; 75°C, 120 s; +42°C 240 s	RCT	6 months	There was no significant difference in VAS among groups with RFT at 70° or 75°C, with or without PRF, but the combination of PRF and RFT helped eliminate postoperative complications	NA

Kosugi [30] (2014)	Japan	89	ITN	30/59	50 Hz, 0.1 to 0.3 V	90°C, 180 s	A single-arm retrospective study	7 years	PRF for V3 showed better long-term outcome than those for V2 or V2 + 3	Weakness of masticatory muscles (12.2%), intolerable dysesthesia (4.9%), eye problem without keratitis (4.9%)

Son [33] (2011)	Korea	38	ITN	20/18	50 Hz, 0.05–0.2 V	70, 75, and 80°C for 60 s	A single-arm retrospective study	38.18 ± 7.79 months	Long-term BNI I (71%), BNI II (15.8%), BNI III (7.9%), and BNI IV (5.3%) recurrent pain rate is 28.9%	Dysesthesia (21%), weakness of the pterygoid or masseter muscles (15.8%)

Yao [34] (2016)	China	1354	ITN	672/682	50 Hz, 1 ms, 0.1–0.2	62°C, 180 s 65°C, 180 s; 68°C, 180 s	Prospective cohort study	9 years	68°C is a good choice for RFT of V2/V3 ITN. 65/62°C minimizes the occurrence of complications but yields a higher recurrence rate	Facial numbness at 62, 65, 68°C accounted for 1.79%, 9.81%, 21.7%; corneal hypoesthesia accounted for 0%, 2.97%, 5.74%; masticatory atonia accounted for 0%, 0.68%, 2.55%

Li [20] (2012)	China	60	ITN	23/37	2 Hz, ≥ 2 V; 50 Hz, ≤ 0.5 V	75°C, 120–180 s; 75°C, 240–300 s; 75, 120–180 s; +42°C, 10 min	RCT	1 year	PRF combined with RFT to the gasserian ganglion can achieve comparable pain relief to those who receive RFT alone	At the same temperature, a longer exposure time results in more severe dysesthesia. Although the combined treatment resulted in more severe dysesthesia immediately, the instance rate was comparable to those who receive RFT alone

Yao [36] (2016)	China	56	ITN	31/29	50 Hz, 1 ms, 0.1–0.2	62°C, 180 s; 62°C, 180 s; +42°C, 10 ms 8 min	Prospective cohort study	3 years	PRF after RFT results in decreased recurrence of V1 TN, reduced numbers of corneal hypoesthesia, shortened recovery time, and increased HRQoL scores	Corneal hypoesthesia accounted for 39.29% and 10.71%; facial numbness was observed in 10.7% and 7.1% at RFT and RFT + PRF group. RFT + PRF group recovered more rapidly

Koning [37] (2013)	Netherlands	25	ITN	12/13	50 Hz, < 0.5 V; 2 Hz, > 0.4 V	65–70°C, 60–180 s	A single-arm retrospective analysis	20–43 months	A lower sensory stimulation threshold during treatment was associated with better patient satisfaction, improved pain relief, and trended toward more hypesthesia	Facial hypesthesia (56%), dryness of the eye (20%), and masseter muscle weakness (12%)

Huang [38] (2016)	China	80	ITN	26/54	NA	V1: 68°C, 2 min; V1 + V2/V3: 70–85°C	A single-arm retrospective study	3 months	The pain-free rate was 98.75% at 3 months	Numbness of the skin in the forehead area (93.8%), disappeared corneal reflex (1.25%), and decreased corneal reflex (12.5%)

Elawamy [39] (2017)	Egypt	43	ITN	19/24	2 Hz, ≥ 0.5–1 V; 50 Hz, 0.3 V	42°C, 4 Hz, 45 V, 10 min; 75°C, 270 s; 42°C 10 min; +60°C, 270 s	RCT	2 years	The best results were observed in the RFT + PRF group, followed by the RFT group and then the PRF group	Anesthesia dolorosa (8.33%), masseter muscle weakness (33.33%), and severe numbness (41.67%) recorded in the CRF group

Erdine [40] (2007)	Turkey	40	ITN	21/19	2 Hz, ≥ 0.1–1.5 V; 50 Hz, 0.1–0.5 V	70, 60 s; 42°C, 45 V, 120 s	RCT	3 months	Pain recurrence rate of PRF group was 100% at 3 months after the procedure	CRF group: mild hypoesthesia and paresthesia (100%), anesthesia dolorosa (5%)

BNI I: the pain disappeared completely, requiring no drugs; BNI II: mild pain, no medication required; BNI III: moderate pain, medication required for complete control; BNI IV: moderate pain, medication required but incomplete control; BNI V: severe or unrelieved pain. PRF: pulsed radiofrequency; RFT: radiofrequency thermocoagulation; VAS: visual analogue scores; ITN: idiopathic trigeminal neuralgia; STN: secondary trigeminal neuralgia.
